# Hyperspectral imaging and artificial intelligence to detect oral malignancy – part 1 - automated tissue classification of oral muscle, fat and mucosa using a light-weight 6-layer deep neural network

**DOI:** 10.1186/s13005-021-00292-0

**Published:** 2021-09-03

**Authors:** Daniel G. E. Thiem, Paul Römer, Matthias Gielisch, Bilal Al-Nawas, Martin Schlüter, Bastian Plaß, Peer W. Kämmerer

**Affiliations:** 1 Department of Oral and Maxillofacial Surgery, Facial Plastic Surgery, University Medical Centre Mainz, Augustusplatz 2, 55131 Mainz, Germany; 2 International Scholar and Adjunct Associate Professor, Department of Oral and Maxillofacial Surgery, School of Dentistry, Kyung Hee University, Seoul, South Korea; 3 School of Technology – Geoinformatics and Surveying, Institute for Spatial Information and Surveying Technology, University of Mainz - University of Applied Science, Mainz, Germany

**Keywords:** Sensoring, Sensors, Future medical, Machine learning, Artificial intelligence, Non-invasive, Non-contact

## Abstract

**Background:**

Hyperspectral imaging (HSI) is a promising non-contact approach to tissue diagnostics, generating large amounts of raw data for whose processing computer vision (i.e. deep learning) is particularly suitable. Aim of this proof of principle study was the classification of hyperspectral (HS)-reflectance values into the human-oral tissue types fat, muscle and mucosa using deep learning methods. Furthermore, the tissue-specific hyperspectral signatures collected will serve as a representative reference for the future assessment of oral pathological changes in the sense of a HS-library.

**Methods:**

A total of about 316 samples of healthy human-oral fat, muscle and oral mucosa was collected from 174 different patients and imaged using a HS-camera, covering the wavelength range from 500 nm to 1000 nm. HS-raw data were further labelled and processed for tissue classification using a light-weight 6-layer deep neural network (DNN).

**Results:**

The reflectance values differed significantly (*p* < .001) for fat, muscle and oral mucosa at almost all wavelengths, with the signature of muscle differing the most. The deep neural network distinguished tissue types with an accuracy of > 80% each.

**Conclusion:**

Oral fat, muscle and mucosa can be classified sufficiently and automatically by their specific HS-signature using a deep learning approach. Early detection of premalignant-mucosal-lesions using hyperspectral imaging and deep learning is so far represented rarely in in medical and computer vision research domain but has a high potential and is part of subsequent studies.

**Supplementary Information:**

The online version contains supplementary material available at 10.1186/s13005-021-00292-0.

## Background

The detection of pathological tissue changes at the macroscopic and microscopic level is one of the foundations of any diagnosis of disease. However, the starting point for any microscopic examination and assessment is the collection of tissue samples. Depending on the aetiology and the affected body region, the precision of diagnosis often differs depending on the invasiveness of sample collection. For example, the gold standard for diagnosis of suspicious oral lesions still consists of incision biopsy with subsequent histopathological examination.

However, this yields disadvantages such as invasiveness and cost intensity. Aiming for a less or non-invasive diagnosis, different procedures (e.g. ^1^brush biopsy [1–3], ^2^*in-vivo* staining with toluidine blue or 5-aminolevulinic acid or the use of ^3^tissue autofluorescence [4, 5]) have been developed during the last years, showing sensitivities of ^1^91 and ^3^91% (77 to 97%) as well as specificities of ^1^91 and ^3^58% (22 to 87%) [6] and providing limited applicability to multifocal lesions with an increased false negative rate [7]. The abovementioned methods have not shown a demonstrable improvement in early cancer detection which is reflected in a consistently high incidence rate of advanced oral squamous cell carcinoma (OSCC). A prerequisite for microscopic assessment of malignancy is the recognition of the physiological status and discrimination against different types of tissue from and to each other. HSI is a promising non-invasive and non-ionizing technique that supports rapid acquisition and analysis of diagnostic information in several fields of clinical medicine [8–13]. For life sciences, the various applications of HSI (monitoring of wound healing [14], perfusion monitoring of microvascular flaps [15], assessment on *in-sano* resection of oesophageal and oropharyngeal carcinomas [16, 17]) have already been successfully demonstrated. HSI includes conventional and spectroscopic methods to obtain both spatial and spectral image information far beyond > 740 nm [18]. Each pixel is assigned a specific vector of radiation values which depends on the chemical material composition of the corresponding localized pixel. This results in large amounts of data, which, however, enables automated tissue recognition through the implementation of machine learning (ML) as an outstanding method that enables researchers to recognize patterns and regularities in increasingly complex data automatically. Modern approaches like deep learning (DL) enlarge origin procedures of ML and move to more complex modelling. As a result, the explicit formulation of rules for high dimensional data can be avoided [19]. DL has become a popular tool in medical data analysis by attaining great achievements in tasks like tissue classification [20, 21] or cancer segmentation [22, 23]. Rapid developments in computer hardware and algorithms have accelerated the success of DL, typically implemented with deep neural networks (DNN) architectures [24, 25]. In medicine DL assists in analysing HSI as a result of the promising non-contact, optical image modality [26–29]. Since we believe that the differentiation of pathological tissue changes, similar to the assessment of blood parameters, is only possible on the basis of a “healthy” standard, the aim of this study was, for the first time, to create a representative HSI data collection of healthy human fat, muscle and oral mucosa, which will serve as a reference library for the assessment of pathological tissue conditions by processing their spectral characteristics with deep learning methods.

## Methods

### Tissue samples

In this prospective, non-randomized experimental study, human excess tissue samples were intraoperatively taken and scanned via HSI. The study was approved by the local ethic committee of Rhineland-Palatinate (registration number: 2020–14,952) and was conducted in accordance with the protocol and in compliance with the moral, ethical and scientific principles governing clinical research as set out in the Declaration of Helsinki of 1975 as revised in 1983. The tissue types examined were transverse muscles, fat and mucosa from oral sites (cheek, vestibule, floor of mouth as well as hard and soft palate). These are the tissue structures most frequently exposed during surgical procedures in the head and neck area. Tissue samples that were macroscopically damaged by the use of bipolar and/or monopolar caustics were excluded from the study. To avoid measurement errors due to blood residues, the tissue samples were washed in a 0.9% saline solution before the examination.

### Hyperspectral cube processing

**Fig. 1:**
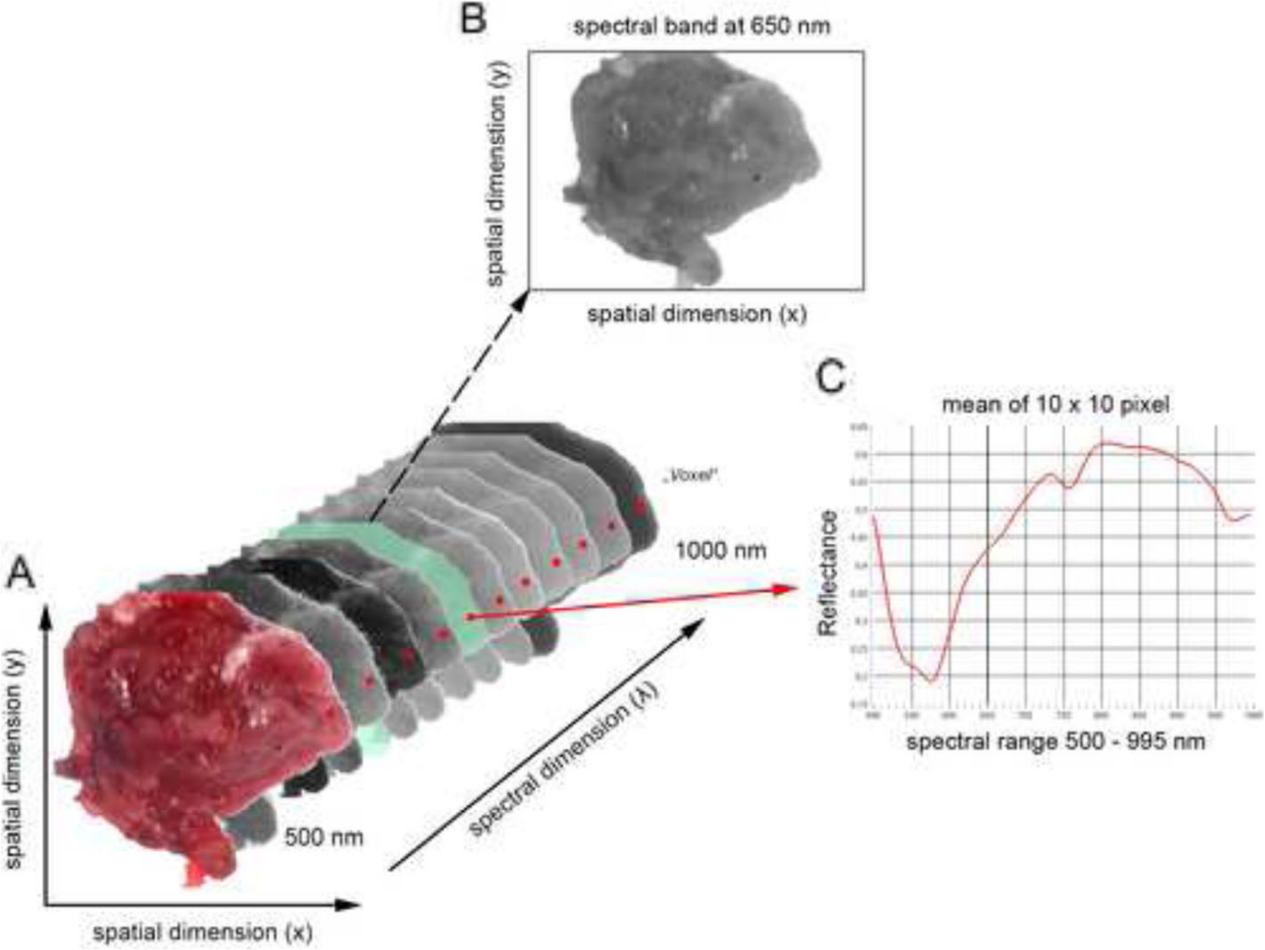
Sample of striated muscle of spatial and spectral dimension (**A**), the isolated two-dimensional grayscale image at 650 nm (**B**), and the reflectance of a selected region of interest (red dot) from 500 nm to 995 nm (**c**)

**Fig. 2:**
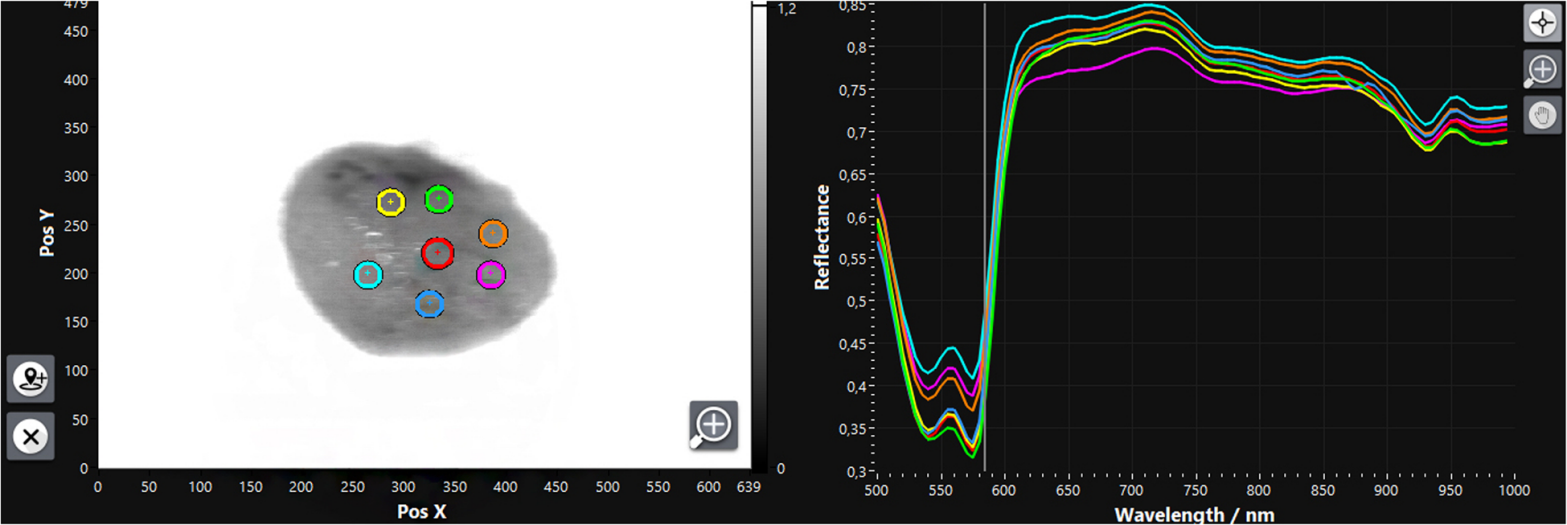
Example of a fat tissue sample with a two-dimensional grey scale image at 585 nm and manually placed (coloured circles) regions of interest (top left), as well as the mean reflectance (coloured lines) as line chart at different wavelengths (top right)

Briefly, HSI is based on the assessment of contiguous spectra (i.e. light of different wavelengths) individually re-emitted by molecules, whereby the molecule-specific re-emitted wave spectrum is generated on the basis of the light spectrum of the halogen spotlights initially emitted for examination. The HS cubes were acquired using a TIVITA Tissue system (Diaspective Vision GmbH, Pepelow, Germany), composed with a 120 W halogen illumination source and a radiometrically calibrated 32-bit complementary metal-oxide semiconductor spectrometer capturing images at a resolution of 480 × 640 pixels. Spatial resolution of the CMOS sensor is 22 μm. The hyperspectral cube contains 100 spectral bands, ranging from 500 nm to 1000 nm with a 5 nm sampling interval and illustrates just a tissue sample belongs to one class. In order to provide training data, 316 tissue samples from 174 patients in total were taken, scanned via HSI, inspected histologically and assigned to the examination classes fat, muscle and oral mucosa. The hyperspectral images for the inspected and classified tissue samples were normalized at each wavelength. For accurate and reproducible measurements, the standard measuring distance was 47.5 cm, ensured by two separate indicator lasers (red laser crosshairs and green laser dot) in an overlapped position in which the green laser dot lies in the centre of the red crosshairs (App. 1.) For image analysis, the camera-specific software package (TIVITA™ Suite) was used [14]. After that, overexposure effects were removed and the arithmetic mean of 7 to 8 manually positioned circular regions of interest (ROIs) with a radius of 5 pixels, each distributed across the tissue sample, were calculated to keep the local proximity (Fig. 1). The arithmetic mean corresponded to the recorded wavelength-specific reflectance values of the ROIs per tissue sample. A radius of 5 pixels has proven to be suitable for placing at least five ROIs on the specimens’ surface. ROIs were placed at different positions whereby its number depended to the surface geometry with ROIs along the border and at least one ROI in the specimens’ centre whenever possible to achieve a homogeneous distribution of the measured area (Fig. 2). The ROIs, named numeric patches, are distributed equally over the tissue sample. To further increase the amount of data, each individual ROI per tissue sample was evaluated as an individual sample in the sense of data augmentation (image subdivision). The classification in this study was conducted using a deep neural network built from scratch using H2O Flow (H2O.ai, version 3.32.1.1, for Microsoft Windows, Open source). For running the experiments, a high-performance notebook operating on Windows 10 Professional with 16GB of RAM and an NIVIDA Quadro T1000 GPU was used.

### Histology

**Fig. 3:**
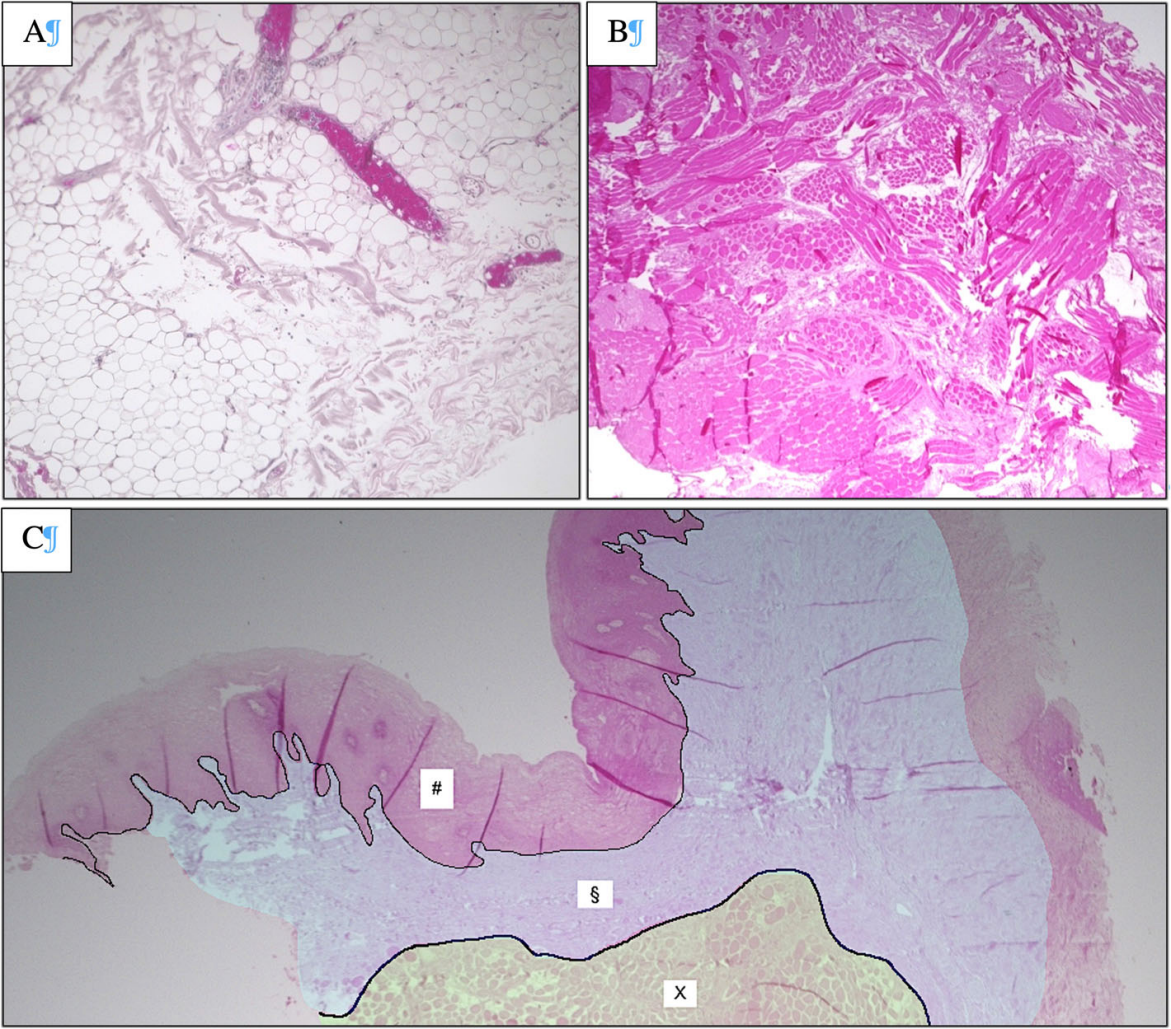
Histological section of a fat sample from the cheek (**A**), as well as a muscle sample (**B**) and a mucosa sample sample (**C**) from the floor of the mouth (stratified squamous keratinised epithelium (#), lamina propria (§-light blue), frontal cut of striated muscle fibres (X-light green)) H.E. staining

After HSI was performed on the tissue samples, the samples were prepared for histological evaluation. For this purpose, the samples were fixed in formalin, embedded in paraffin, cut in 5 μm steps using a microtome, applied to slides and stained with haematoxylin and eosin (H&E). After slide digitization, they were examined with regard to the tissue composition (i.e. proportion of fat and musculature in the total tissue sample, as well as lack of inflammation). Samples containing other tissue types (e.g. muscle on mucosa specimen), which accounted for > 5% of the examined preparation surface, as well as inflammatory modified mucosa samples, were excluded from the evaluation due to result distortion. Examples of histological sections are shown in Fig. 3.

### Statistics

Raw data sets were saved in Excel® sheets (Microsoft Corporation, Redmond, USA) and subsequently transferred into SPSS Statistics® (version 23.0.0.2, MacOS X; SPSS Inc., IBM Corporation, Armonk, NY, USA). Data were expressed as median (MD), mean (M), standard deviation (SD±), minimum (min), maximum (max) and standard error of the mean (SEM). Normal distribution was checked using non-parametric Kolmogorov-Smirnov test (KS test) and results were analysed for statistical significance by the use of analysis of variance (ANOVA ^(#)^), unpaired non-parametric Mann-Whitney U tests = ^($)^ and students’ t-test = ^(^*^)^. *P*-values of ≤0.05 were termed significant. For a proof of principle study sample size calculation is not practicable, thus this study is in accordance to other published proof of concept works dealing with similar group sizes [30, 31]. Line charts were used for illustration purposes.

### Neural network

The processed training data were split in ratio 85:8:7 (training, validation, test), while training patches used to fit the networks weights. In addition, validation patches intended to optimize hyperparameter and test patches used for evaluation purpose of the fitted model. The data split procedure was performed considering leave-patient-out approach. This feed-forward neural network was trained stepwise in mini-batches of 64 numeric patches with a patient-ID based stratified cross-validation paradigm for 4000 epochs using early stopping techniques for plateauing. A uniform adaptive distribution was used to set initial weights. To improve generalisation, a dropout rate of 30% was applied after first and 20% after second and third hidden layer. Each neuron was activated using rectified linear unit (ReLU). Training was performed using balanced classes and an adaptive learning rate for stochastic gradient descent optimization [32] with momentum of 0.99 and a smoothing factor equal to 1 × 10^− 8^. Furthermore, L1 and L2 regularisation terms of 5 × 10^− 3^ each for reducing the cross-entropy loss was set up as shown in Fig. 6. Before training, hyperparameter such as neural network architecture, dropout quantity, activation function, learning rate related parameters, regularization terms and batch size were adjusted with systematic grid search technique by taking care of the overfitting gap between training and validation loss. Every epoch, the validation performance was evaluated but shuffling was disabled caused by higher losses. The final softmax-layer predicts inputs with respect to the highest probability of each class. Testing is done after training and validation loss converges equally. As plotted in Fig. 6, the model generalises in a fast manner. The deep neural network performance was evaluated on the optimal checkpoint applied on fully independent test data to calculate accuracy (Acc; $Accuracy=\frac{TP\ \left( true\ positive\right)+ TN\left( true\ negative\right)}{Total\  No. of\ patches}$), specificity (Spec; $Specificity=\frac{TN}{TN+ FP}$) and sensitivity (Sens; $Sensitivity=\frac{TP}{TP+ FN}$).

## Results

### Ex-vivo fat, muscle and mucosa - spectral signatures

**Fig. 4:**
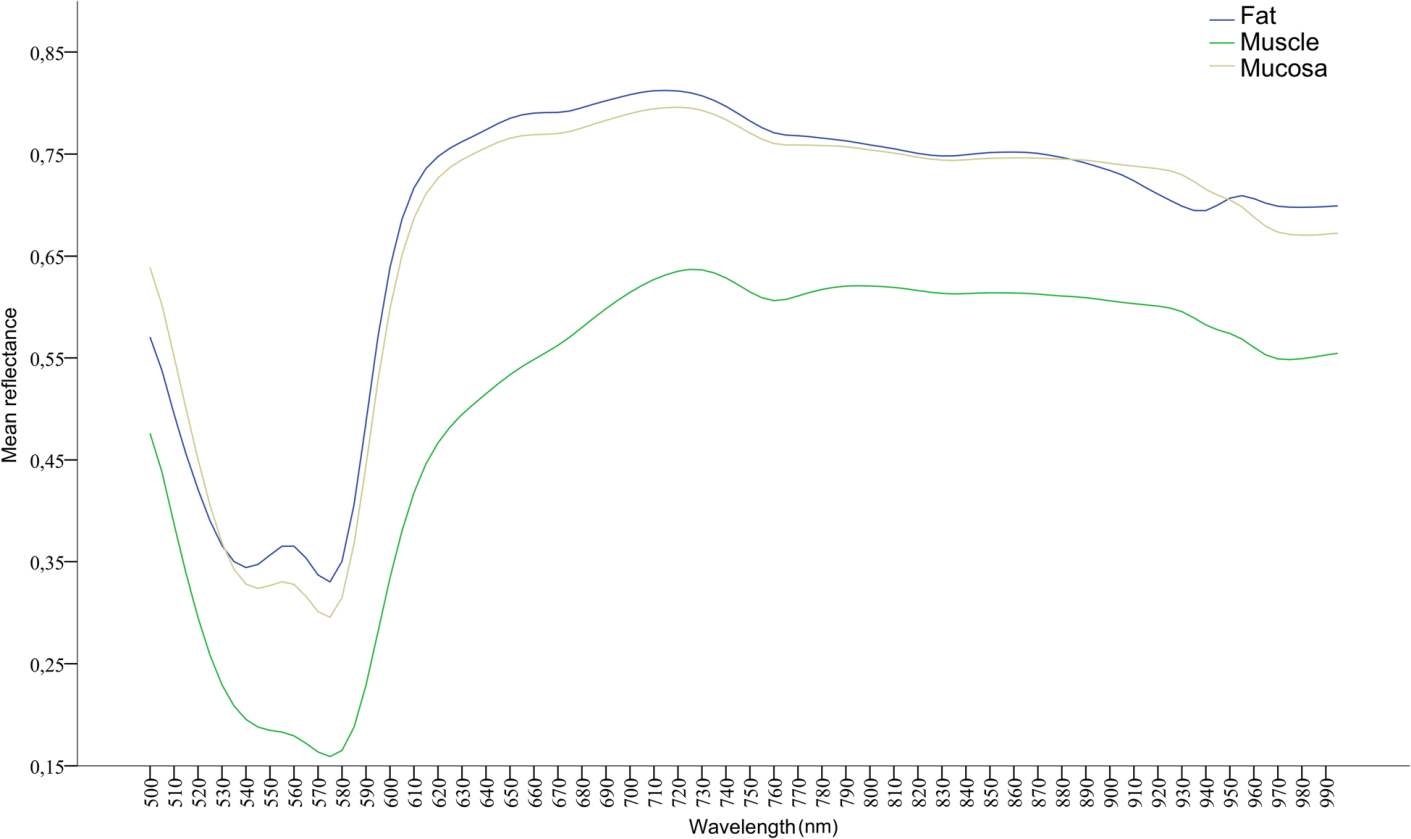
Normalized spectral signatures that were averaged between all patients / tissue samples that were included in this study

The spectral signature for each class obtained from the hyperspectral imaging processing is plotted in Fig. 4.

When comparing fat (group-1) and muscle (group-2), as well as muscle (group-2) and mucosa (group-3) the mean reflectance values differed significantly at all wavelengths (*p* < .001). In contrast, the spectral signatures of fat (group-1) and mucosa (group-3) appeared much more similar in the graphical overview (Fig. 4), but also differed significantly at most wavelengths (500 nm to 520 nm, 545 nm to 790 nm, 910 nm to 940 nm and 950 nm to 995 nm (p < .001^$^*)). Detailed information on mean values, standard deviations and individual significances are available on request.

### Neural network

**Fig. 5:**
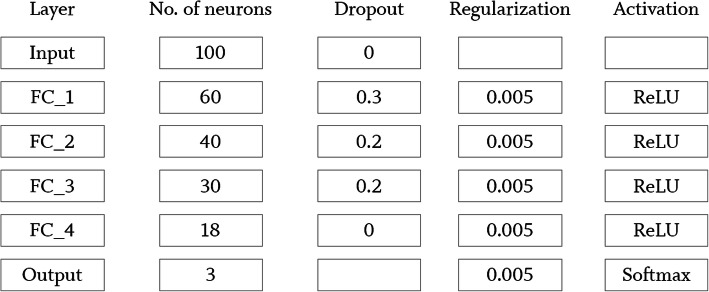
Neural network architecture implemented for medical classification of numeric patches with H2O Flow

The amount of training data and the distribution among classes are shown in Table 1. As shown in Fig. 5, a light-weight architecture was implemented for the studies.

**Table 1:** Number of tissue samples from 174 patients included in this study. The total of numeric patches as data source for deep neural network classification obtained from each sample class is also given

Class	No. of tissue samples (n)	Total patches (m)
Fat	97	681
Muscle	101	707
Mucosa	118	826

### Evaluation

Classification scores were calculated using values reported in Table 2 based on common equations [20]. Interestingly, a comparatively high classification error between musculature and mucosa was found (error 0.21). Table 3 shows the evaluation results.

**Table 2:** Confusion Matrix for tissue sample classification using cross validation approach. Actual classes are reported in rows and predicted classes in columns. Misclassified patches of muscle as mucosa show the unresolved deficit of our model so far

	Predicted Class			
Actual Class	Fat	Muscle	Mucosa	Error
Fat	74	0	8	0.10
Muscle	3	68	15	0.21
Mucosa	1	9	92	0.10
Total	83	79	108

**Table 3:** Results of evaluation using independent test data with a size of 21 tissue samples patches equally distributed at the checkpoint after 254 epochs with lowest loss of 0.1321 as shown in Fig. 6. Values are reported as class-dependent classification scores (Acc = accuracy, Spec = specificity, Sens = sensitivity)

Class	Samples	Acc	Spec	Sens
Fat	7	0.95	1.00	0.86
Muscle	7	0.86	0.79	1.00
Mucosa	7	0.81	0.93	0.57

## Discussion

**Fig. 6:**
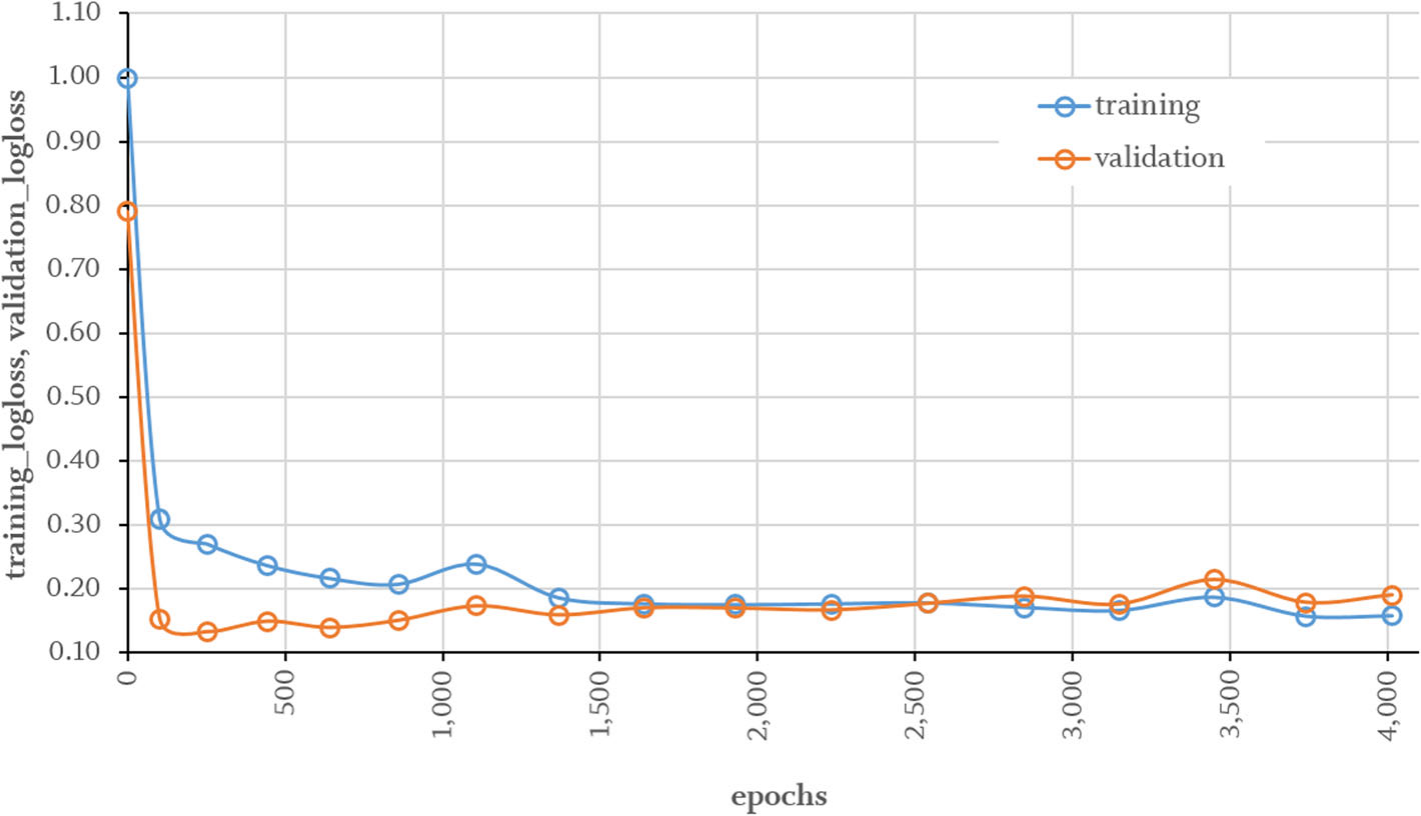
Training and validation losses for 4000 epochs. Validation losses are reported as average across every 5 folds

Although non-invasive examination methods (e.g. brush biopsy or tissue autofluorescence) have been developed to monitor oral potentially malignant disorders (OPMD), histopathological examination still represents the diagnostic gold standard for lesion monitoring. HSI is a non-invasive, non-contact optical wide-field modality that holds the potential to sense tumours in varying depth using visible spectrum (VIS) and near infrared (NIR) light and therefore to improve OPMD monitoring, early oral cancer diagnosis and reduce cancer-related mortality and morbidity [20, 33]. To process the extensive amount of spectra-spatial data cube information efficiently and automatically, the use of DL methods is suitable. Knowledge of the spectral characteristics of its main components (oral mucosa, muscle and fat) is essential for the assessment of complex, pathologically altered oral mucosa. This study presents a method to classify 316 fresh surgical ex-vivo human oral tissue samples’ reflectance values into fat, muscle and mucosa, based on HSI data of a representative number of samples. This light-weight deep learning (DL) approach achieved an overall accuracy score over 87% in an ordinary and time-saving manner, but with commonly used optimization techniques. Together with a lot more patient’s metadata and a hyperspectral database of many samples from different individuals, the clinical use for non-invasive, automated assessment of oral mucosal changes would be a conceivable and tangible approach. Therefore, the data should simultaneously serve as a kind of hyperspectral reference library for future applications such as the in-vivo examination of chronic inflammatory oral diseases, the intraoperative assessment of surgical safety margins or the intraoperative assessment of lymph nodes when deciding to include higher lymph node levels. Using a light-weight 6-layer deep neural network with only 10,445 parameters trained about 4000 epochs, we can distinguish tissue samples in fat, muscle and oral mucosa with a class accuracy over 80% each. Though, the presented solution provided fully independent test data [20] and this study’s training was not affected by overfitting, however the optimisation potential can be increased even further as shown in Fig. 6. A limitation of this approach is the high-dimensional feature space with about 100 (500 to 1000 nm) wavelengths. Further experiments with significantly reduced number of features based on feature selection and dimensionality reduction processes present a more complex problem the research group is currently working on. By gaining more sample data, the generalisation potential of the deep learning approach and the numerical discrepancy between sensitivity and specificity regarding muscle and mucosa as shown in Table 3 could improve. However, the false-positive classification of muscle and mucosa in the test data set (error 0.21) compared to fat versus muscle or mucosa (error 0.10) proved to be relatively high (Table 2). One explanation for this would be the anatomically determined increased contamination of the mucosal tissue with musculature, which was mainly found in the area of the cheeks and soft palate mucosa.

Preliminary results of this proof of concept study demonstrate the ability of deep learning methodology for discriminating between hyperspectral tissue samples. Future studies are going to deal with classification between healthy, dysplastic and cancerous tissue samples based on Convolutional Neural Network (CNN) approaches with non-pre-processed hyperspectral cube data.

## Conclusions

The processing of hyperspectral tissue data by a neural network allows the automated classification of tissue samples with increasing model accuracies. Our research deals with the classification of healthy oral fat, muscle and mucosa by using of HS-reflectance values and thus differs to alternative approaches build upon HS-images and CNNs. With an accuracy of > 80% our model in comparison to Halicek et al. [18] lacks accuracy but relies on numeric patches characterises fast training and feed-forward phase. In order to eliminate the accuracy gap, future studies will emphasize convolutional building blocks and image data. Provided that sufficient hyperspectral training data of dys- and anaplastic mucosa samples are available, the combination of hyperspectral imaging and deep learning can thus represent a promising method for a real time non-invasive assessment of oral mucosal changes.

## Supplementary Information

**Additional file 1: Appendix 1.** The figure shows the measurement situation with laser marking.

## Data Availability

The datasets used and/or analysed during the current study are available from the corresponding author on reasonable request.

## Abbreviations

Acc: Accuracy
CNN: Convolutional Neural Network
DL: Deep Learning
DNN: Deep Neural Networks
H&E: Haematoxylin and Eosin
HS: Hyperspectral
HSI: Hyperspectral Imaging
OSCC: Oral Squamous Cell Carcinoma
m: Mean
Max: Maximum
MD: Median
Min: Minimum
ML: Machine Learning
NIR: Near Infrared
OPMD: Oral Potentially Malignant Disorders
ReLU: Rectified Linear Unit
ROIs: Regions of Interest
SD: Standard Deviation
Sens: Sensitivity
Spec: Specificity
VIS: Visible Spectrum
