# Supplementary figures and images for: Hyperspectral imaging and artificial intelligence to detect oral malignancy – part 1 - automated tissue classification of oral muscle, fat and mucosa using a light-weight 6-layer deep neural network
Source: Head Face Med. 2021 Sep 3;17:38. doi: 10.1186/s13005-021-00292-0 (PMC8414848; doi:10.1186/s13005-021-00292-0)

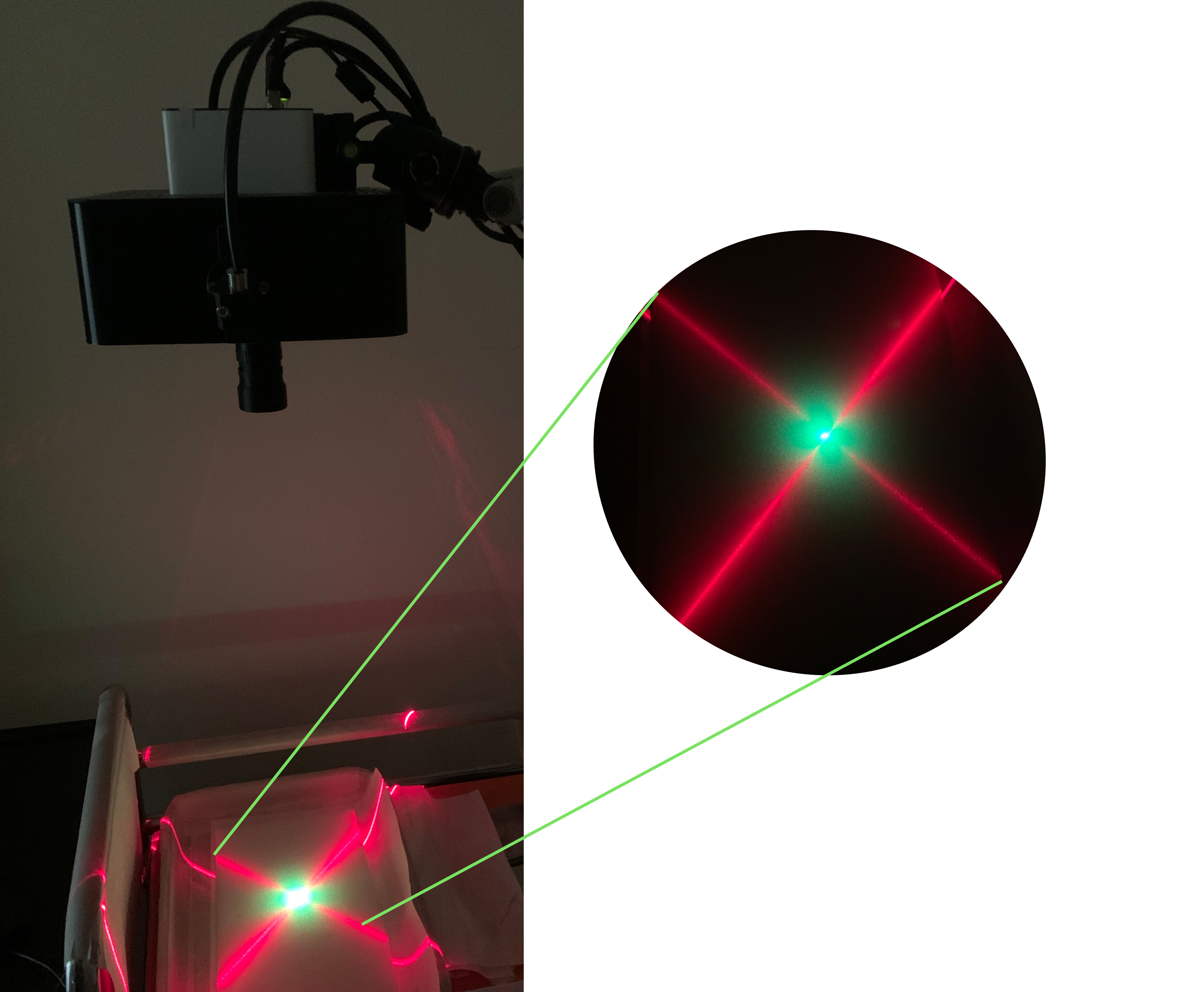

Supplement: Supplementary file 1 — Additional file 1: Appendix 1. The figure shows the measurement situation with laser marking. [file 13005_2021_292_MOESM1_ESM.jpg]
